# Mitochondria-targeted antioxidant preserves contractile properties and mitochondrial function of skeletal muscle in aged rats

**DOI:** 10.18632/oncotarget.5783

**Published:** 2015-09-22

**Authors:** Sabzali Javadov, Sehwan Jang, Natividad Rodriguez-Reyes, Ana E. Rodriguez-Zayas, Jessica Soto Hernandez, Tanja Krainz, Peter Wipf, Walter Frontera

**Affiliations:** ^1^ Department of Physiology, School of Medicine, University of Puerto Rico, San Juan, PR, USA; ^2^ Department of Chemistry, University of Pittsburgh, Pittsburgh, PA, USA; ^3^ Department of Physical Medicine and Rehabilitation, Vanderbilt University School of Medicine, Nashville, TN, USA

**Keywords:** aging, skeletal muscle, single fiber contractility, mitochondrial ROS, XJB-5-131, Gerotarget

## Abstract

Mitochondrial dysfunction plays a central role in the pathogenesis of sarcopenia associated with a loss of mass and activity of skeletal muscle. In addition to energy deprivation, increased mitochondrial ROS damage proteins and lipids in aged skeletal muscle. Therefore, prevention of mitochondrial ROS is important for potential therapeutic strategies to delay sarcopenia. This study elucidates the pharmacological efficiency of the new developed mitochondria-targeted ROS and electron scavenger, XJB-5-131 (XJB) to restore muscle contractility and mitochondrial function in aged skeletal muscle. Male adult (5-month old) and aged (29-month old) Fischer Brown Norway (F344/BN) rats were treated with XJB for four weeks and contractile properties of single skeletal muscle fibres and activity of mitochondrial ETC complexes were determined at the end of the treatment period. XJB-treated old rats showed higher muscle contractility associated with prevention of protein oxidation in both muscle homogenate and mitochondria compared with untreated counterparts. XJB-treated animals demonstrated a high activity of the respiratory complexes I, III, and IV with no changes in citrate synthase activity. These data demonstrate that mitochondrial ROS play a causal role in muscle weakness, and that a ROS scavenger specifically targeted to mitochondria can reverse age-related alterations of mitochondrial function and improve contractile properties in skeletal muscle.

## INTRODUCTION

The progressive age-related decline in skeletal muscle mass and strength, known as sarcopenia, involves changes in several metabolic pathways [1, 2]. Alterations in energy metabolism associated with impaired mitochondrial function and oxidative capacity are central events in aging myocytes [3, 4]. The main alterations observed in mitochondria with aging include: i) high mitochondrial ROS (mtROS) generation associated with increased damage to proteins, lipids, and mitochondrial DNA (mtDNA), ii) activation of stress response pathways, and iii) decreased expression of mtDNA-encoded proteins associated with a progressive loss of mitochondrial respiratory function [5-7]. As a result, mitochondria of old muscles generate less ATP and cannot provide adequate energy for muscle actions contributing to sarcopenia. Human studies have correlated age-related declines in O_2_ consumption [8] and ATP synthesis [3] with reduced mitochondrial mass [9] and oxidative phosphorylation activity [10]. Importantly, mitochondrial dynamics significantly change with age because sustained mitochondrial elongation induces senescence-associated phenotypic changes in the cell, resulting in low mitochondrial membrane potential, high mtROS production, and mtDNA damage [11]. Giant, dysfunctional mitochondria with highly interconnected networks and ultrastructural abnormalities produce more ROS and have a low autophagic activity that further ablates the mitochondrial quality control in aged cells [12, 13].

While accumulation of mtROS plays a causal role in the pathogenesis of sarcopenia, the contributions of changes in the electron transfer chain (ETC) activity and increases in mtROS to age-related muscle weakness continues to be intensely debated. Furthermore, there is no pharmacologic treatment to reverse mitochondrial deficits in the elderly. Targeted expression of mitochondrial catalase preserved structure and function of mitochondria and extended lifespan [14, 15] that was associated with reduced insulin resistance and muscle dysfunction in mice [15]. Treatment with the mitochondria-targeted peptide antioxidant SS-31 attenuated mitochondrial degeneration, improved age-related skeletal muscle performance [16] and protected the diaphragm against mechanical ventilation-induced myofiber atrophy and contractile dysfunction [17]. Likewise, a derivative of plastoquinone (SkQ) exerted multiple anti-aging effects, prevented ROS accumulation, and was especially effective during the early and middle stages of aging [18]. These studies provide a proof-of-principle for a causal role of mtROS in sarcopenia and suggest the use of targeted antioxidant interventions as a promising and highly effective therapeutic intervention.

**Figure 1:**
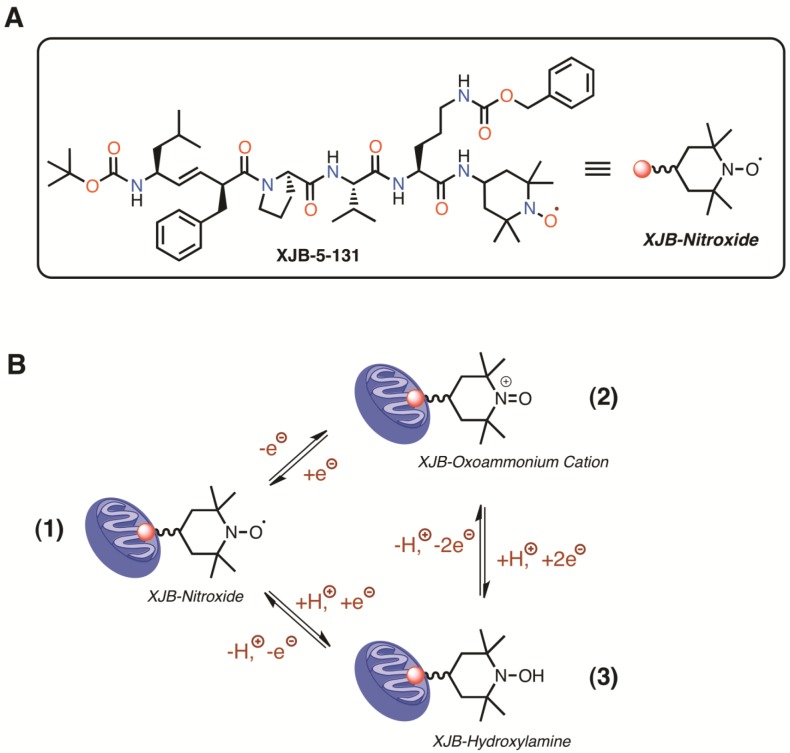
Structure (A), and redox properties of XJB (B) XJB is effectively enriched in mitochondria, where the nitroxide moiety acts as an ROS and electron/radical scavenger. Conversion of the nitroxide function into other oxidation states is reversible and can contribute to catalytic ROS turnover through the following interactions (B). The one-electron reduction of the nitroxide (1) in the XJB molecule generates a hydroxylamine (2), which can undergo a two-electron oxidation to the oxoammonium cation (3). Single-electron reduction of the oxoammonium cation regenerates the nitroxide. Therefore, all of these N-O oxidation states can equilibrate as a function of the redox state of the environment and the presence of glutathione and NAD(P)H [44]. Superoxide radical anion, as well as reactive nitrogen species, react with all XJB N-O oxidation states and participate in the redox cycle (B) [45]. The nitroxide radical is also a superb single-electron and radical acceptor, and can quench species originating from hydrogen atom abstraction by ROS, among other pathways [46].

A derivative of the antibiotic gramicidin S, XJB-5-131 (XJB), is a recently developed antioxidant that targets mitochondria and provides mtROS and electron scavenging capacity by virtue of its conjugation to a 4-amino-2,2,6,6-tetramethylpiperidinooxy (4-Amino-TEMPO) moiety [19]. The structure and redox properties of XJB are summarized in Figure 1. The major antioxidant capacity of XJB is likely to involve the scavenging of electrons leaking from the ETC, rather than being caused by an SOD-like mechanism [20]. XJB significantly reduced the disease phenotype and improved mitochondrial function of striatal synaptosomes in a mouse model of Huntington's disease [21]. Mouse embryonic cells pretreated with XJB demonstrated reduced apoptosis and enhanced cell survival [22]. Intravenous administration of XJB prevented ileal mucosal barrier dysfunction and tissue ischemia associated with significantly prolonged survival from hemorrhagic shock [23] and protected against global cerebral ischemia-reperfusion injury [24] in rats. Our recent studies demonstrated that hearts of XJB-treated aged rats were resistant to ischemia-reperfusion injury, as evidenced by improved post-ischemic recovery of cardiac function and reduced cell death. Also, they exhibited higher mitochondrial respiration rates than untreated age-matched counterparts [25].

In this study, we determined the possible beneficial effects of XJB on contractile properties and mitochondrial ETC activity of skeletal muscle in aged rats. The results demonstrated that aged animals treated with XJB possess high muscle contractile activity associated with improved mitochondrial function and decreased protein oxidation.

## RESULTS

### Mitochondria-targeted ROS scavenging does not affect gravimetric parameters in aged rats

**Figure 2:**
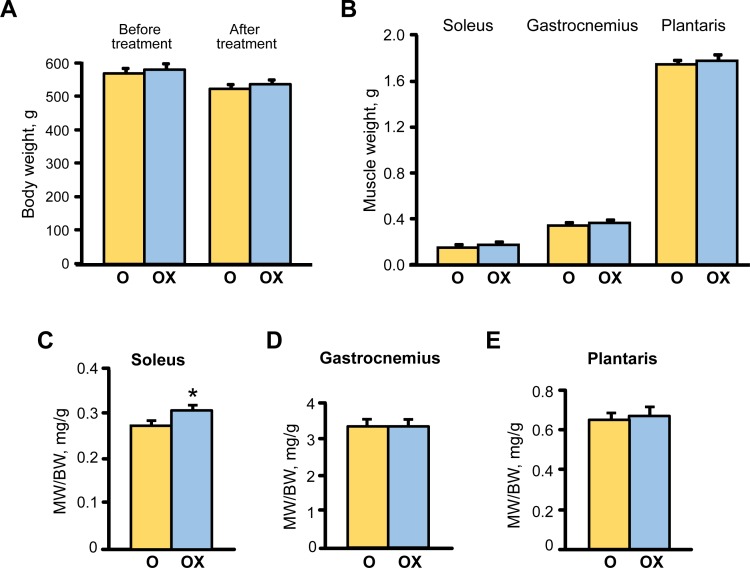
The effect of XJB on gravimetric parameters in aged XJB-treated (OX) and untreated (O) rats **A.**, Body weight (BW); **B.**, Muscle (MW); **C.**, MW/BW for soleus; **D.**, MW/BW for the gastrocnemius, and **E.**, MW/BW for plantaris. *P < 0.05 O *vs*. OX.

Analysis of gravimetric parameters showed that XJB treatment did not affect the body weight (BW) of aged rats that demonstrated a slight but equal (~8%) decrease in untreated and treated groups by the end of the treatment period (Figure 2A). Equally, plantaris and gastrocnemius weights were not affected by XJB, although soleus weight was increased by 14.9% (*P* < 0.05) in the XJB-treated group (Figure 2B). Consequently, the muscle-to-body weight ratio remained unchanged for plantaris and gastrocnemius but was a 10.3% (*P* < 0.05) lower for the soleus muscle in the treated group (Figure 2C-2E). No animal died during the treatment. These findings indicate that, in general, treatment of aged rats with XJB has minimal effect on BW and skeletal muscle weights.

### XJB treatment has no effect on the muscle fiber size but improves single fibre contractile properties in aged skeletal muscle

**Figure 3:**
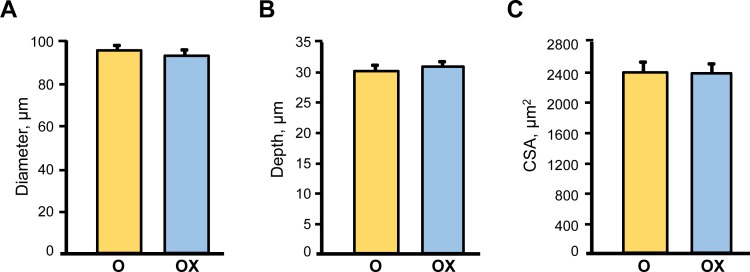
Single muscle fibre morphological characteristics in aged XJB-treated (OX) and untreated (O) rats Diameter **A.**, depth **B.** and cross-sectional area (CSA, **C.**) were measured in single muscle fibers isolated from the gastrocnemius muscle of XJB-treated or untreated old rats.

**Figure 4:**
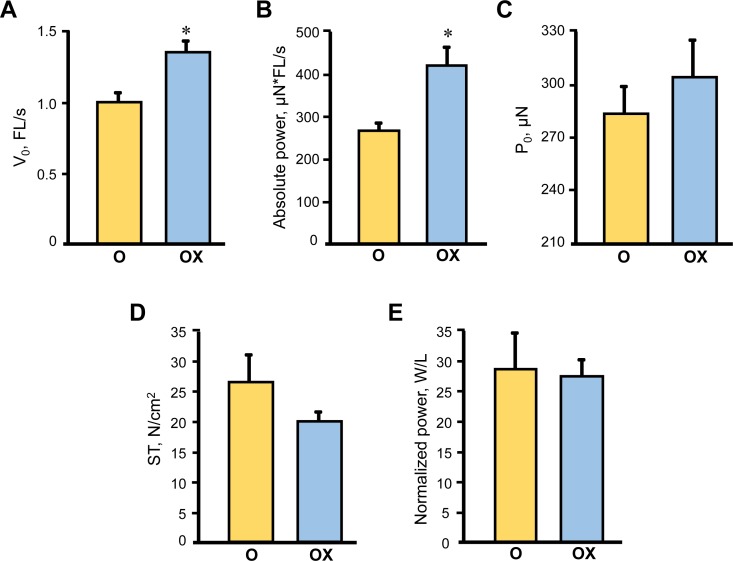
Single muscle fibre contractile properties in aged XJB-treated (OX) and untreated (O) rats Contractile properties were measured in single muscle fibers isolated from the gastrocnemius muscle of XJB-treated or untreated old rats. Maximum unloaded shortening velocity (V_0_, A), absolute power calculated as the product of velocity and force (B), maximum force (P_0_, C), specific force calculated as P_0_ normalized to CSA (ST, D), and normalized power (E) calculated as absolute power normalized to CSA. *P < 0.01 OX *vs*. O.

Due to the labor intensity and sample size needed for the single fibre experiments we assessed single muscle fibre size and contractile properties only in the gastrocnemius muscle isolated from XJB-treated/untreated old rats. Treatment with XJB had no effect on single fibre dimensions (diameter, depth, and cross-sectional area) (Figure 3). Analysis of contractile properties demonstrated that XJB significantly increased the maximal unloaded shortening velocity (Vo) and absolute power that were 35% (*P <* 0.01) and 58% (*P <* 0.01) higher, respectively, when compared with the untreated group (Figure 4A, 4B). These statistical comparisons accounted for the fact that several fibres from the same animal were included in the analysis. Intriguingly, differences between groups became not significant when force and absolute power were adjusted for fibre size (specific force, ST) and fibre length (normalized power), respectively (Figure 4D, 4E). Collectively, these results demonstrate that treatment with XJB has no effect on the muscle fiber size but improves single fibre contractile properties in aged skeletal muscle.

### XJB prevents oxidative damage to proteins in skeletal muscle

**Figure 5:**
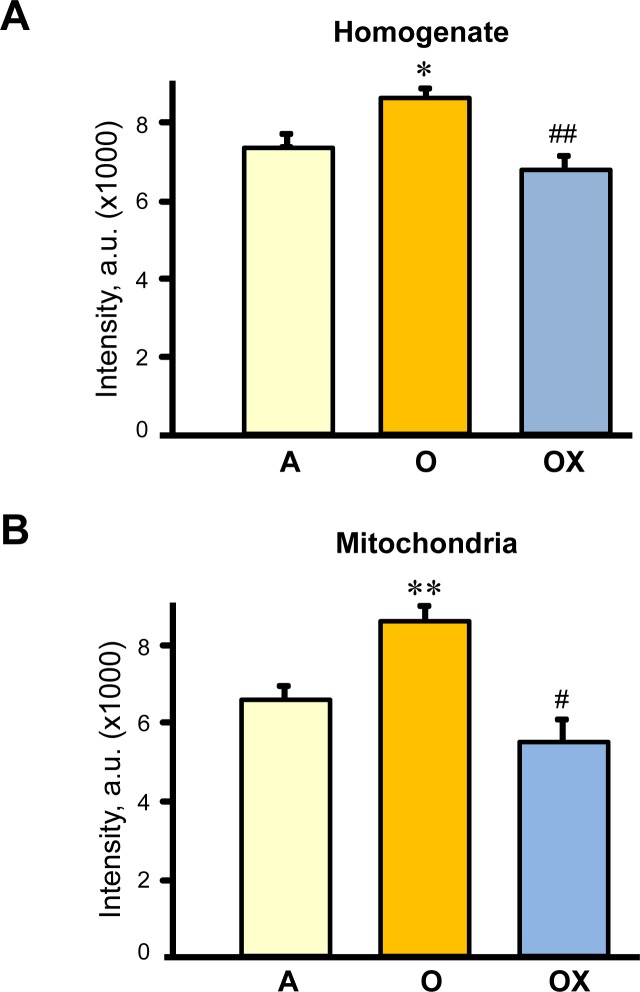
Protein carbonylation levels in homogenate (A) and (B) mitochondria isolated from the gastrocnemius of adult (A), and XJB-treated (OX) or untreated (O) old rats Protein carbonylation determined by western blotting using anti-dinitrophenyl antibodies. Levels of carbonylation were calculated as the sum of all band intensities for each lane after subtraction of non-specific background signal. *P < 0.05, **P < 0.01 O *vs.* A, ^#^ *P* < 0.05, ^##^ *P* < 0.01 OX *vs*. O.

Next, we determined whether XJB reduces protein carbonylation, as a marker of oxidative damage, in skeletal muscle of aged rats. Muscle homogenate and mitochondria isolated from gastrocnemius were used for analysis of carbonyl levels. Carbonyl levels in the muscle homogenate and mitochondria of aged animals increased by 17% (*P <* 0.05) and 31% (*P <* 0.01), respectively, compared to adult (6-months old) rats (Figure 5A, 5B). As expected, aging led to more oxidative damage to mitochondrial than homogenate proteins. Treatment with XJB reduced protein oxidation in both fractions. These data suggest that improvements observed with contractile properties of gastrocnemius may be associated with reduced oxidative stress in aged skeletal muscle.

### The beneficial effects of XJB on muscle contractility are associated with increased activity of ETC complexes

**Figure 6:**
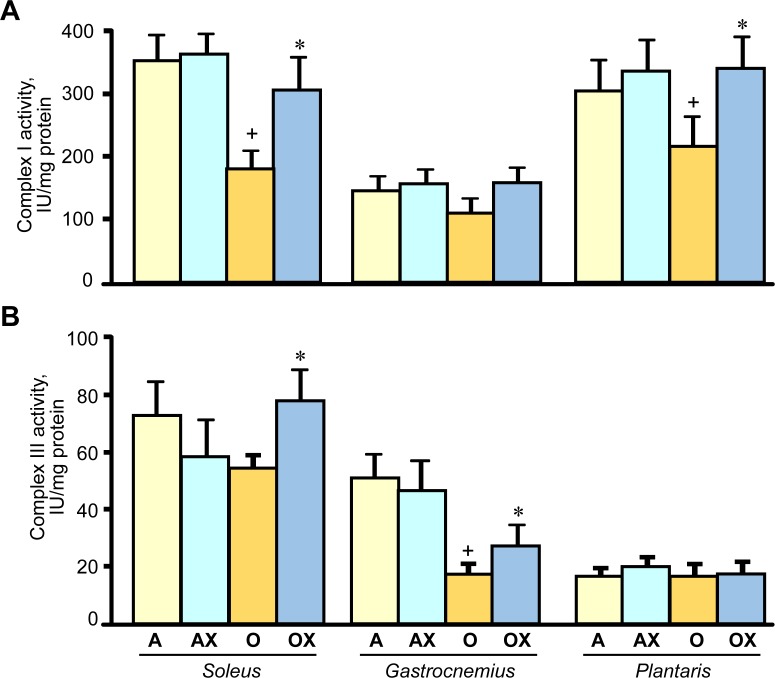

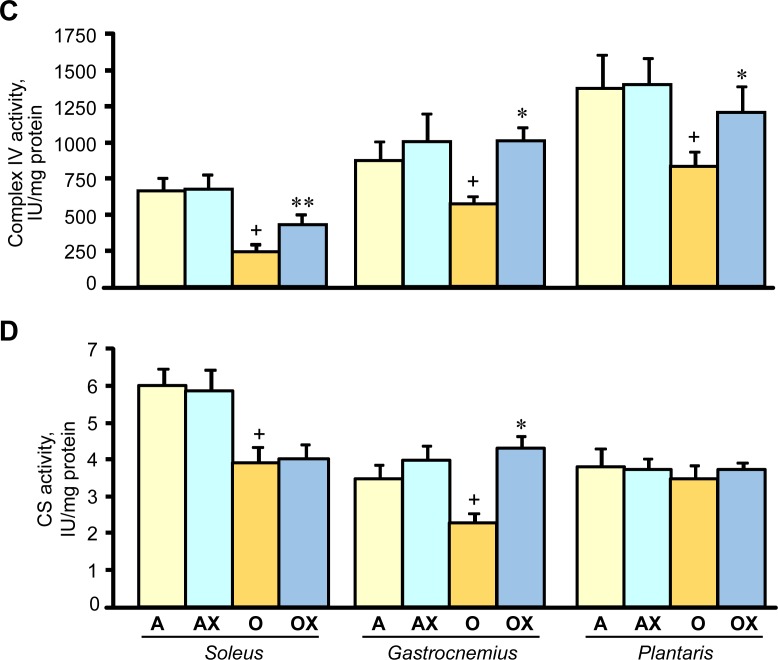
Enzymatic activity of the ETC complexes I (A), III (B), and IV (C), and citrate synthase (CS, D) in mitochondria isolated from soleus, gastrocnemius, and plantaris of XJB-treated (OX) or untreated (O) old rats The activity of complexes is expressed in IU per mg mitochondrial protein. *P < 0.05, **P < 0.01 OX *vs*. O.

**Figure 7:**
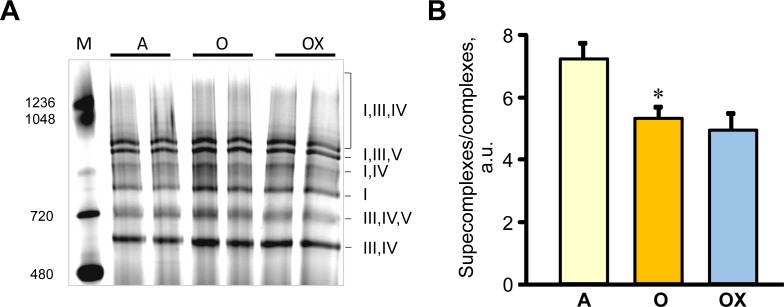
ETC supercomplexes levels measured by blue native gel electrophoresis in gastrocnemius mitochondria of adult (A), and XJB-treated (OX) and untreated (O) old rats **A.** Representative blue native gel image demonstrates supercomplexes containing complexes I, III, IV, and V, and individual complex I. **B.** Quantitative data represent the ratio of supercomplexes to complex I calculated as sum of supercomplexes I+III+IV, I+III+V and I+IV to individual complex I levels.

**Figure 8:**
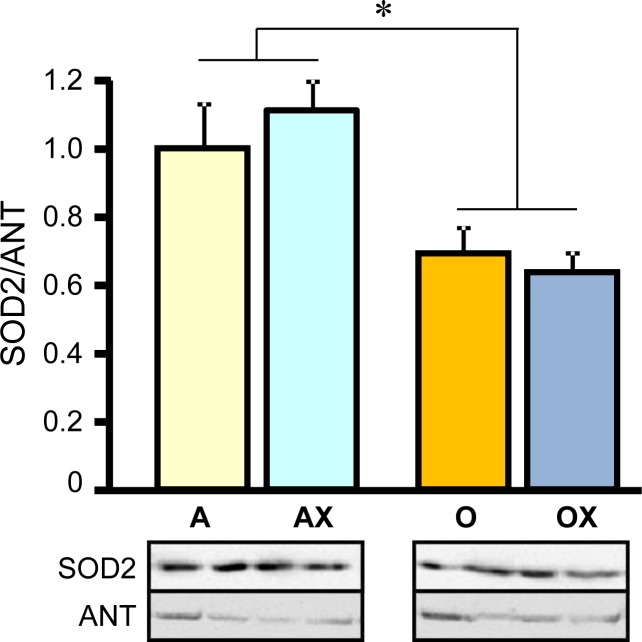
Protein levels of superoxide dismutase 2 (SOD2) in gastrocnemius mitochondria isolated from adult (A, AX) and old (O, OX) rats treated or untreated with XJB Bottom panels show representative immunoblots (two per each group) with SOD2 and adenine nucleotide translocase (ANT) antibodies. Data were normalized to ANT (a mitochondrial housekeeping protein) and expressed as a fold change compared to the adult XJB-untreated (A) group. *P < 0.05 O and OX *vs*. A and AX.

Furthermore, we determined the activity of ETC complexes in mitochondria isolated from all three muscles. We revealed no differences between XJB-treated and untreated adults rats on the activity of ETC complexes and citrate synthase (Figure 6A-6D). In old rats, XJB exerted diverse effects on different muscles for ETC complexes I, III, and IV. Complex I activity in soleus and plantaris mitochondria of XJB-treated rats increased by 69% (*P* < 0.05) and 60% (*P* < 0.05), respectively, compared to untreated counterparts (Figure 6A). The ROS scavenger also increased complex I activity in the gastrocnemius (by 41%, *P* = N.S.); however, the difference was not statistically significant. Treatment with XJB induced a 39% (*P* < 0.05) and 59% (*P* < 0.05) increase of the complex III activity in soleus and gastrocnemius, respectively, with no effect on plantaris mitochondria (Figure 6B). The activity of complex IV was markedly improved in all three muscles isolated from XJB-treated animals that were an 83% (*P* < 0.01), 70% (*P* < 0.05) and 44% (*P* < 0.05) higher in soleus, gastrocnemius and plantaris, respectively, compared with mitochondria from untreated old rats (Figure 6C). Notably, there were no differences in citrate synthase activity between treated and untreated groups for all three muscles, indicating that mitochondrial mass was not affected by the ROS scavenger (Figure 6D). Also, analysis of ETC supercomplexes in gastrocnemius mitochondria revealed a 25% (*P* < 0.01) decrease in aged rats compared with adult counterparts. However, there were no differences between XJB-treated and untreated aged rats (Figure 7A, 7B). Likewise, XJB had no effect on protein levels of superoxide dismutase (SOD2), a mitochondrial isoform of SOD (Figure 8). These data are consistent with our previuos findings (20) and demonstrate that the antioxidant effects of XJB occur through its capacity to scavenge electrons leaking from uncoordinated electron carriers rather than the SOD-like activity. Altogether, these data demonstrate that XJB treatment improves ETC complex activity without an effect on mitochondrial mass and ETC supercomplex levels in aged skeletal muscles.

## DISCUSSION

The present study provides new evidence that *in vivo* treatment of old rats with the recently developed potent mitochondria-targeted antioxidant XJB: i) improves some contractile properties of single skeletal muscle fibres, ii) increases the activity of ETC complexes in skeletal muscle mitochondria, and iii) has no detrimental effect on the muscle-to-body weight ratio and single fibre size. These data provide strong experimental evidence that mtROS play a causal role in aging-related muscle weakness, and that the treatment of aged animals with a ROS/electron scavenger specifically targeting mitochondria may delay sarcopenia and improve contractility in skeletal muscle.

Several lines of evidence in both experimental and clinical studies have supported a central role of mtROS in aging, and the mitochondrial free radical theory emphasises the importance of developing new pharmacological compounds that would specifically accumulate in mitochondria and target mtROS to attenuate aging (reviewed in [26-28]). Targeted overexpression of catalase in mitochondria resulted in a significant lifespan extension in mice that provided a direct evidence for the role of mtROS in longevity [14, 15]. We found that XJB preserved mitochondrial function (ETC activity) that was associated with improved contractility in single muscle fibres of old rats. Our data are also consistent with previous studies showing that treatment of accelerated aging Ercc1(−/Δ) mice with XJB attenuated age-related intervertebral disc degeneration and improved intervertebral disc cell metabolism [29].

Several studies have revealed a causal link between enhanced mitochondrial function and improved muscle contractility. The flavanol (-)-epicatechin, a component of dark chocolate, increased expression of ETC complexes and mitochondrial biogenesis that was associated with significantly enhanced fatigue resistance in isolated skeletal muscles and improved treadmill performance in young mice [30]. Likewise, high mitochondrial biogenesis and cytochrome c expression induced by administration of quercetin was associated with significantly improved exercise capacity in young mice [31]. We have previously reported [25] a direct mtROS scavenging effect of XJB in cultured H9c2 cells that are used widely as an *in vitro* cellular model for both skeletal and cardiac muscle. Pre-treatment of these cells with XJB was associated with preserved mitochondrial membrane potential and increased cell survival and significantly reduced mtROS induced by hydrogen peroxide, DMNQ (ROS inducer) and antimycin A (complex III inhibitor). Interestingly, unchanged citrate synthase activities in treated and untreated animals suggested that the improvements in mitochondrial function with XJB were not simply due to increased mitochondrial mass (Figure 6D). These observations are consistent with previous studies that investigated anti-aging effects of a mitochondria-targeted peptide with mtROS scavenging capacity, SS-31. Age-related declines in mitochondrial ATP production, coupling of oxidative phosphorylation (P/O), and cell energy state (PCr/ATP) were rapidly reversed after SS-31 treatment with no effect on mitochondrial content as evidenced by unchanged protein expression of ETC complexes in old mice [16]. Notably, like SS-31, uptake of XJB into mitochondria is very high (up to 600-fold versus the cytosolic fraction) and largely independent of the mitochondrial membrane potential [20, 32]. In addition, accumulation of the scavenger does not affect the mitochondrial membrane potential in cardiac myocytes [25].

The anti-aging effects of XJB and maintenance of skeletal muscle contractility presumably occur due to improved mitochondrial ATP synthesis and energy transfer in the ETC. This preserved ETC activity due to XJB-induced reduction of mtROS may abolish mtDNA damage. Mitochondrial biogenesis and function is regulated by the network of transcription factors that control nuclear and mitochondrial genes encoding mitochondrial proteins [33]. MtDNA encodes 13 of the approximately 80 proteins involved in ETC complexes and mtDNA damage induced by mtROS overproduction alters mitochondrial gene expression leading to defective protein synthesis. Therefore, scavenging of mtROS may improve the ETC activity presumably through prevention of mtDNA damage in aged hearts. In support of this mechanistic hypothesis, previous studies revealed the ability of XJB to attenuate oxidative DNA damage in a mouse model of Huntington's disease [21]. Notably, prevention of mtDNA may be one of the pathways that mediate beneficial effects of XJB. Along with the conservation of mtDNA, prevention of protein (i.e. myosin) oxidation (Figure 5) may play a critical role in the anti-aging effects of XJB. This could contribute to the preservation of muscle fiber contractility. In addition, XJB interacts closely with cardiolipin, a unique mitochondria-specific phospholipid, and very efficiently prevents its oxidation [20, 24]. Cardiolipin plays an essential role in the assembly and stabilization of ETC supercomplexes. Aging reduced the protein level of supercomplexes in skeletal muscle (Figure 7), and these findings are consistent with previous studies [34]. However, XJB treatment had no effect on the supercomplex levels in aged skeletal muscle. These data indicate that the beneficial effects of XJB on ETC complexes are not mediated through assembling supercomplexes, at least, in gastrocnemius. Further studies are required to clarify the effect of XJB on ETC supercomplexes in different skeletal muscles as well as mitochondrial subpopulations (subsarcolemmal and interfibrillar mitochondria).

In conclusion, our findings provide strong evidence that reductions in mtROS emission by XJB improve the mitochondrial ETC activity and contractility in aged skeletal muscles. These protective effects might be due to increased ATP production, and decreased oxidation of proteins, lipids, and nucleic acids. Development of a new generation of ROS scavengers that primarily target mtROS has a clinical significance for the treatment of age-related diseases, and for delaying the degenerative processes closely associated with aging. The beneficial effects observed with XJB in attenuating the muscle weakness in aged rats indicate the potential importance of this ROS and electron scavenger as a new therapeutic agent.

## LIMITATIONS OF THE STUDY

One of the limitations is that the studies were conducted only in male rats. Gender differences in muscle contractility and mitochondrial function could play a significant role in the effect of XJB. In addition, our study involves the treatment of aged rats with XJB for one month, which might be insufficient to exert more remarkable effects on the mitochondrial function and skeletal muscle contractility. High sensitivity of muscle fibers to low-temperature freezing did not allow the analysis of the muscle contractility in all groups.

## MATERIALS AND METHODS

### Animals

Male adult (5-month old, n = 17) and aged (29-month old, n = 19) Fischer Brown Norway (F344/BN) rats were purchased from Charles River (NIA NIH colony at Charles River, Kingston, NY). All experiments were performed according to the protocol approved by the University Animal Care and Use Committee and conform to the Guide for the Care and Use of Laboratory Animals published by the US National Institutes of Health (NIH Publication No. 85-23, revised 1996).

### Experimental protocols

Animals were randomly assigned to the following four groups: adult (A), adult+XJB (AX), aged (O), and aged+XJB (OX). XJB was dissolved in 0.5 mL of sunflower seed oil (Sigma-Aldrich, St. Louis, MO) and administered intraperitoneally 3 times per week (3 mg/kg body weight) for four weeks. Rats in untreated groups (A and O) were injected an equal volume of sunflower oil (vehicle) without XJB. The body weights of the animals were measured weekly. After four weeks of treatment, animals were sacrificed, and skeletal muscles were isolated, weighed, and used for analysis of contractile properties and isolation of mitochondria.

### Single fibre extraction and permeabilization

The gastrocnemius muscle was dissected from rats and placed in iced relaxing solution (in mM; 4 Mg-ATP, 1 free Mg^2+^, 20 imidazole, 7 EGTA, 14.5 creatine phosphate, and sufficient KCl to adjust the ionic strength to 180). The pH was adjusted to 7.0. The muscles were separated into bundles of fibres and chemically skinned for 24 h in skinning solution (relaxing solution containing 50% (v/v) glycerol) at 4°C. The chemically skinned bundles were stored at ­20°C up to 4 weeks until experimental analysis or treated for long-term storage at ­80°C using sucrose, a cryoprotectant.

### Analysis of single fibre dimensions

Single fibre dimensions were determined as previously described [35]. Briefly, the skinned fibre bundles were placed in relaxing Brij solution containing 0.5% Brij-58 (polyoxyethylene 20 cetyl ether; Sigma-Aldrich, St. Louis, MO) for 30 min at 4°C. Then, the bundles were transferred to relaxing solution for the isolation of single fibres. Each fibre was transferred to an experimental apparatus (1400A Permeabilized Fiber Test System, Aurora Scientific Inc., Ontario, Canada) and mounted on the stage of an inverted microscope (Olympus IX71, Tokyo, Japan). The fibre was attached between connectors leading to a force transducer (Model 403A, Aurora Scientific Inc., ON, Canada) and a direct-current torque motor (Model 315C, Aurora Scientific, ON, Canada). Immediately, the fibre segment was submerged in relaxing solution at 15°C to proceed with morphological measurements. The sarcomere length (set to 2.75-2.85 μm) and the fibre's diameter, depth, and length were measured using the Video Sarcomere Length Image Analysis System (Aurora Scientific Inc., ON, Canada). Fibre cross-sectional area (CSA) was calculated using width and depth of the fibre, assuming an elliptical circumference and corrected for the 20% swelling that follows skinning [36, 37].

### Determination of contractile properties in single fibres

The contractile properties of single fibers were determined with the slack test procedure [38, 39]. Maximal unloaded shortening velocity (V_0_) was measured as the change in length of the fibre *vs*. change in time. Briefly, after 15 sec in a relaxing solution, the fibre was transferred to a low Ca^2+^-EGTA solution for another 15 sec to reach steady tension and to preserve the regularity of cross-striations. Then, the fibre was submerged in an activating solution (relaxing solution with 10^−4.5^ M Ca^2+^). Once the maximum force (plateau) was reached, a slack (shortening step) was imposed to drop the force rapidly to zero. The time required for the fibre to develop tension again from the imposed slack was measured. The fibre was relaxed and slowly re-extended to its original length. This procedure was repeated with different amplitudes of slacks (5, 7.5, 10, 12.5, and 15% of the total fibre segment length). After the procedure was completed, each fibre was stored in a sample buffer containing 0.5 M Tris-HCl, pH 6.8, 15% glycerol, 1% SDS, and 0.5% bromophenol blue, 10 mM dithiothreitol, and 2 μM leupeptin at −80°C until the electrophoretic analysis was performed.

For each fibre, the relationship between the time required for force re-development and the slack length was plotted using least-squares regression. The slope corresponds to the V_0_ of the fibre. Maximum active force (P_0_) was calculated as the difference between the total tension in activating solution (pCa 4.5) and the resting tension measured in the same segment while in relaxing solution. The specific force was calculated as P_0_ normalized to CSA. Absolute power was calculated as the product of velocity and force and normalized power as absolute power normalized to CSA.

### Isolation of mitochondria

Mitochondria were isolated from all 3 skeletal muscles using minor modifications of the method described previously [40]. Briefly, muscles were removed immediately after sacrifice and rinsed in ice-cold saline solution. Then they were finely minced into small pieces and incubated in the solution containing 10 mM EDTA and 0.05% trypsin for 15 min. Then the minced muscle samples were centrifuged at 800 *g* for 2 min, and the pellet was homogenized in (1:5 wt/vol) ice-cold isolation buffer containing 300 mM sucrose, 10 mM Tris-HCl, 10 mM KCl, 10 mM EDTA, pH 7.4, and 0.5% BSA using a Polytron homogenizer. The homogenate was centrifuged at 2,000 *g* for 2 min at 4°C. The supernatant was centrifuged at 10,000 *g* for 5 min, and the resultant pellet was washed one more time at 10,000 *g* for 5 min in the isolation buffer containing no BSA. The final mitochondrial pellet was resuspended in the buffer and used for analysis of protein expression and enzyme activities. All procedures were performed at 4°C.

### Enzyme activity of ETC complexes and citrate synthase

Mitochondrial samples were freeze-thawed three times and incubated in hypertonic media (25 mM KH_2_PO_4_, 5 mM MgCl_2_, 0.5 mg/mL BSA) supplemented with 0.55 mg/mL saponin for 30 min at 4°C before use in enzyme analysis to completely destroy mitochondrial membranes and provide access of substrate to ETC complexes. All assays were performed at the Thermo Scientific GENESYS™ 10S UV-Vis spectrophotometer at 30°C as previously described [41].

#### Complex I (NADH-ubiquinone oxidoreductase) activity

The activity of complex I was determined by measuring the decrease in the concentration of NADH at 340 nm [42]. The assay was performed in buffer solution containing 5 mM KH_2_PO_4_, pH 7.5, 5 mM MgCl_2_, 0.24 mM CoQ_1_, 0.5 mM KCN, 1 mg/mL BSA, and 2.5 μg/mL antimycin A. The reaction was initiated by the addition of 20 μM NADH. The enzyme activity was measured for 3 min, and complex I activity was determined by calculating the slope of the reaction in the presence or absence of 2 μg/mL rotenone (complex I inhibitor). The activity of complex I was expressed as nmol oxidized NADH/min per mg protein.

#### Complex III (ubiquinol-cytochrome c reductase) activity

The activity of complex III was determined in buffer solution containing 5 mM KH_2_PO_4_, pH 7.5, 5 mM MgCl_2_, 0.24 mM CoQ1, 0.5 mM KCN, 1 mg/mL BSA, 2 μg/mL rotenone, and 0.12 mM cytochrome c (oxidized form). The reaction was initiated by the addition of 0.02 mM NADH. The activity of ubiquinol: cytochrome c reductase was measured as the rate of reduction of cytochrome c at 550 nm with 580 nm as the reference wavelength. The activity of complex III was expressed in nmol reduced cytochrome c/min per mg protein.

#### Complex IV (cytochrome c oxidase) activity

The activity of complex IV was determined by measuring the oxidation of cytochrome c at 550 nm in a buffer solution containing 50 mM KH_2_PO4, pH 7.5, 60 mM KCl, 2 μg/mL rotenone, and 0.03 mM cytochrome c previously reduced with dithiothreitol. The reaction was initiated by the addition of reduced cytochrome c and monitoring the slope in the presence or absence of 0.20 mM KCN (complex IV inhibitor). The activity of complex IV was expressed as nmol oxidized cytochrome c/min per mg protein.

#### Citrate synthase activity

The activity of citrate synthase was determined spectrophotometrically by measuring coenzyme A formation at 412 nm as described previously [43]. Enzyme activity was expressed as nmol oxaloacetate/min per mg protein.

### Analysis of SOD2, and ETC supercomplexes

Mitochondrial proteins were separated by SDS-PAGE and immunoblotted with antibodies against SOD2 or adenine nucleotide translocase (Santa Cruz Biotechnology, Santa Cruz, CA). The chemiluminescence signals were visualized at the VersaDoc 3000 Gel Imaging System (Bio-Rad, Hercules, CA). Mitochondrial ETC supercomplexes were analyzed by blue native gel electrophoresis as previously described [34]. Briefly, 60 μg of mitochondrial proteins were dissolved with 50 μL of solubilization buffer (50 mM NaCl, 50 mM imidazole-HCl, 2 mM 6-aminohexanoic acid, 1 mM EDTA) supplemented with 2 μL of 20% digitonin, 0.5 μL protease and phosphatase inhibitor cocktails (Sigma-Aldrich, St. Louis, MO), and 12.5 U of Benzonase. Samples were incubated on ice for 20 min and then centrifuged for 20 min at 20,000 g. Supernatants were collected and mixed with 15 μL of sample buffer (50 mM NaCl, 10% glycerol, 0.001% Ponceau S, 50 mM Tris-HCl, pH 7.2). Blue native gel electrophoresis was conducted as the manufacturer's recommendations (Invitrogen). After electrophoresis, the gels were stained by Coomassie brilliant blue G 250, then scanned with the Odyssey CLx Infrared Imaging System (LI-COR Biosciences, Lincoln, NE). The resulting images were analyzed with ImageJ (NIH).

### Protein carbonylation assay

Protein carbonyls were analyzed as described previously [25]. Briefly, samples of homogenate and mitochondria isolated from the gastrocnemius muscle were derivatized with dinitrophenylhydrazine (DNPH, Sigma-Aldrich, St. Louis, MO) under acid denaturing conditions. Proteins were separated by SDS-PAGE and immunoblotted with anti-dinitrophenyl primary antibodies (Sigma-Aldrich, St. Louis, MO) at 1:1000 dilution. In order to correct for non-specific binding of antibodies, separate acid-denatured but no DNPH-treated protein samples were run in parallel. Carbonyl levels were determined in scanned blots as the sum of all band intensities for each lane after subtraction of non-specific background signals.

### Statistical analysis

The statistical software STATA version 12.0 (STATA Corp, College Station, TX) was used to perform the statistical analysis. Differences between treated and untreated groups were determined by 2-tailed Student's t-test. Also, a linear mixed regression model was employed to examine differences in single fibre properties between treated and untreated groups. Mixed linear models allow the evaluation of the correlation between multiple fibres per each specimen analysed (random effects component of the model). This model assumes that each specimen has its own mean measurement (normal distribution between specimens) and each measurement for each subject of interest is normally distributed around the mean value. Data are presented as means±SEM. Differences were considered to be statistically significant when *P* < 0.05.

## Abbreviations

BW: body weight
CSA: cross-sectional area
ETC: electron transport chain
HW: heart weight
mtROS: mitochondrial ROS
mtDNA: mitochondrial DNA
P_0_: maximum active force
ROS: reactive oxygen species
ST: specific force
V_0_: maximum unloaded shortening velocity
XJB: XJB-5-131 (L-ornithinamide, 1-[(2*S*,3*E*,5*S*)-5-[[(1,1-dimethylethoxy)carbonyl]amino]-7-methyl-1-oxo-2-(phenylmethyl)-3-octen-1-yl]-L-prolyl-L-valyl-*N*^5^-[(phenylmethoxy)carbonyl]-*N*-(2,2,6,6-tetramethyl-1-oxy-4-piperidinyl)-)
